# Potassium doping increases biochar carbon sequestration potential by 45%, facilitating decoupling of carbon sequestration from soil improvement

**DOI:** 10.1038/s41598-019-41953-0

**Published:** 2019-04-02

**Authors:** Ondřej Mašek, Wolfram Buss, Peter Brownsort, Massimo Rovere, Alberto Tagliaferro, Ling Zhao, Xinde Cao, Guangwen Xu

**Affiliations:** 1 UK Biochar Research Centre, School of Geosciences, University of Edinburgh, Crew Building, Alexander Crum Brown Road, Edinburgh, EH9 3FF UK; 2 Scottish Carbon Capture & Storage, School of Geosciences, University of Edinburgh, High School Yards, Infirmary Street, Edinburgh, EH1 1LZ UK; 3 Applied Science and Technology Department, Politecnico di Torino, Corso Duca degli Abruzzi 24, 10129 Torino, Italy; 4 Institute for Site Contamination and Remediation, School of Environmental Science and Engineering, Shanghai Jiao Tong University, 800 Dongchuan Road, Shanghai, 200240 China; 5 Institute of Industrial Chemistry and Energy Technology, Shenyang University of Chemical Technology, No. 11 Street, Economic and Technological Develoment Zone of Shengyang, Shengyang, 102115 China

## Abstract

Negative emissions technologies offer an important tool to limit the global warming to <2 °C. Biochar is one of only a few such technologies, and the one at highest technology readiness level. Here we show that potassium as a low-concentration additive in biochar production can increase biochar’s carbon sequestration potential; by up to 45% in this study. This translates to an increase in the estimated global biochar carbon sequestration potential to over 2.6 Gt CO_2_-C(eq) yr^−1^, thus boosting the efficiency of utilisation of limited biomass and land resources, and considerably improving the economics of biochar production and atmospheric carbon sequestration. In addition, potassium doping also increases plant nutrient content of resulting biochar, making it better suited for agricultural applications. Yet, more importantly, due to its much higher carbon sequestration potential, AM-enriched biochar facilitates viable biochar deployment for carbon sequestration purposes with reduced need to rely on biochar’s abilities to improve soil properties and crop yields, hence opening new potential areas and scenarios for biochar applications.

## Introduction

Technologies for CO_2_ removal from the atmosphere (so called Negative Emission Technologies) will be required to limit the global warming to <2 °C^1,2^. Sequestering carbon in soil in the form of biochar has been discussed for around a decade and has shown great potential as a carbon negative strategy^3–5^. Although uncertainties still exist regarding estimation of the residence time of biochar in soil based on its physical and chemical properties and soil conditions^6^, it is generally accepted that carbon in biochar has a residence time in soil several orders of magnitude greater than the biomass it was produced from^7^. Therefore, many studies use proxies for assessing the amount of stable carbon in biochar^8–10^. Most relevant is the amount of carbon stable after 100 years in soil, the duration typically used to calculate the global warming potential of greenhouse gases^11^. In various articles the carbon sequestration potential of biochar has been calculated to be in the range of 0.7–1.8 Gt CO_2_-C_(eq)_ yr^−1^^3,12,13^.

Biochar is produced as one of the co-products of pyrolysis, which is the thermochemical conversion of biomass in the absence of free oxygen at temperatures above around 350 °C. This process yields three co-products in the form of pyrolysis solids (biochar), pyrolysis liquids (organic acids, phenolic compounds, etc.), and gases (CO, CO_2_, H_2_, CH_4_, C_2_H_4_, etc.)^14,15^. The yield of biochar, and also its carbon stability, are dependent on feedstock and pyrolysis conditions^16,17^.

Increasing the percentage of stable carbon content in biochar is one way of increasing its carbon sequestration potential (the other is increasing biochar yield); this is typically achieved by increasing the severity of the pyrolysis process (higher temperature and longer residence time, which reduces solids yield, increasing C release as gas, leading to higher CO_2_ emissions when burned)^8,18^. In context of carbon capture and storage, however, the increase of the stable carbon yield, i.e. the amount of stable carbon that can be obtained from the same amount of biomass, is more relevant. Increasing the stable carbon yield relative to the parent biomass feedstock is in effect equivalent to reducing the amount of biomass needed to sequester a unit of carbon.

The stability of biochar carbon is related to its condensed aromatic nature^19^, which in turn is affected by the composition of biomass feedstock and by processing conditions, mainly highest treatment temperature (HTT). Besides organic constituents (cellulose, hemicellulose and lignin), biomass also contains inorganic constituents, which content and composition is dependent on the type of biomass, growing location and to some extent the method of harvest and subsequent treatment^20,21^. Previous studies showed that concentrations of certain constituents of the mineral matter, especially alkali metals (AM) and alkaline earth metals (AEM), such as K, Na, Ca, Mg, strongly affect biochar yields^22–26^, with biochar yields increasing with elevated levels of AMs and AEMs due to catalysis of biochar formation. Yet, little is know about the effects of AMs and AEMs on the yield of stable carbon in biochar. In some studies the carbon retention in biochar was increased using additives, though very high additive to biomass ratios (w/w) were used, e.g. in Ren *et al*.^26^ Ca(OH)_2_ in a ratio of 1:9 was applied, in Zhao *et al*.^25^ H_3_PO_4_ was applied in ratios of 0.359:1 and 0.718:1. In this study, we have used an order of magnitude lower loading of a low-cost additive (1 and 2%) which at the same time provides K to plants when resulting biochar is applied to soil.

The potential ability of AMs to catalyse biochar formation together with the fact that potassium is a valuable macronutrient makes the use of AMs as additives in biochar production a potentially attractive proposition. This is especially true, if the AM catalysed biochar has at least similar or better properties in terms of carbon stability as biochar produced without AM doping. Despite extensive research on biochar stability, no systematic investigation of stability of AM catalysed biochar has been reported to date. The research presented in this paper focused on increasing the carbon sequestration potential of biochar derived from the energy crop *Miscanthus giganteus*, by increasing biochar yield and stability using a common, low-cost additive, potassium acetate, and comparing the effects with those of sodium acetate doping.

## Results and Discussion

### Potassium doping increases biochar and stable carbon yield

**Figure 1:**
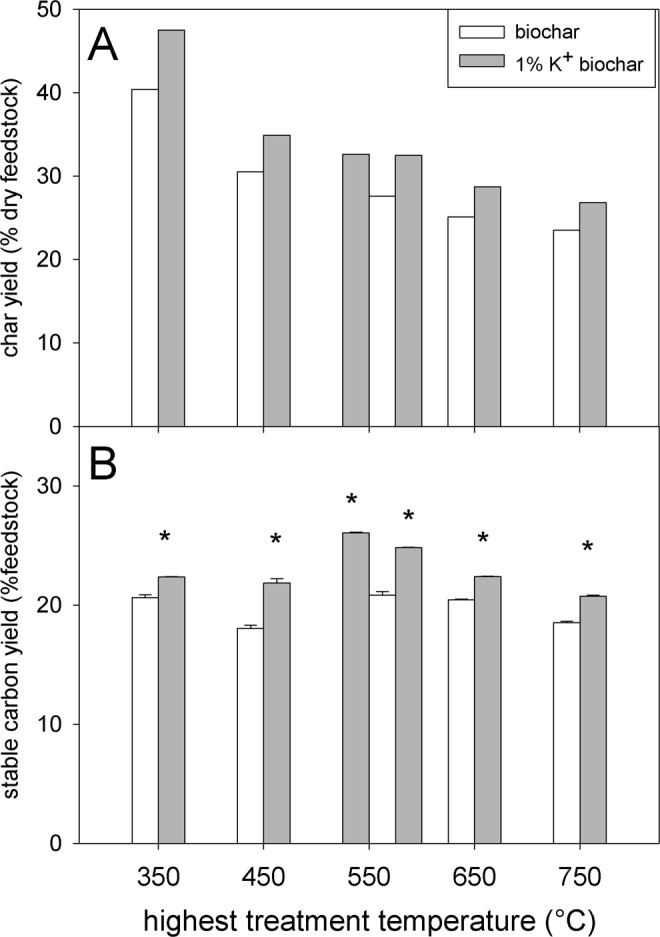
*Miscanthus* biochar yield (**A**; n = 1) and stable carbon yield (**B**; n = 3) of eleven biochar types. Eleven biochar types were produced at five different temperatures with and without 1% K^+^ doping (replicate at 550 °C). Stable carbon yield data is depicted on dry feedstock basis, taking the biochar yield into account (calculations see materials and methods). Error bars in Figure B show one standard deviation. The asterisks indicate statistically significant differences of the 1% K^+^ doping compared to the control, using two-sample, two-sided, equal variances t-tests. No statistical analysis was performed on biochar yield (no replications of pyrolysis itself).

**Figure 2:**
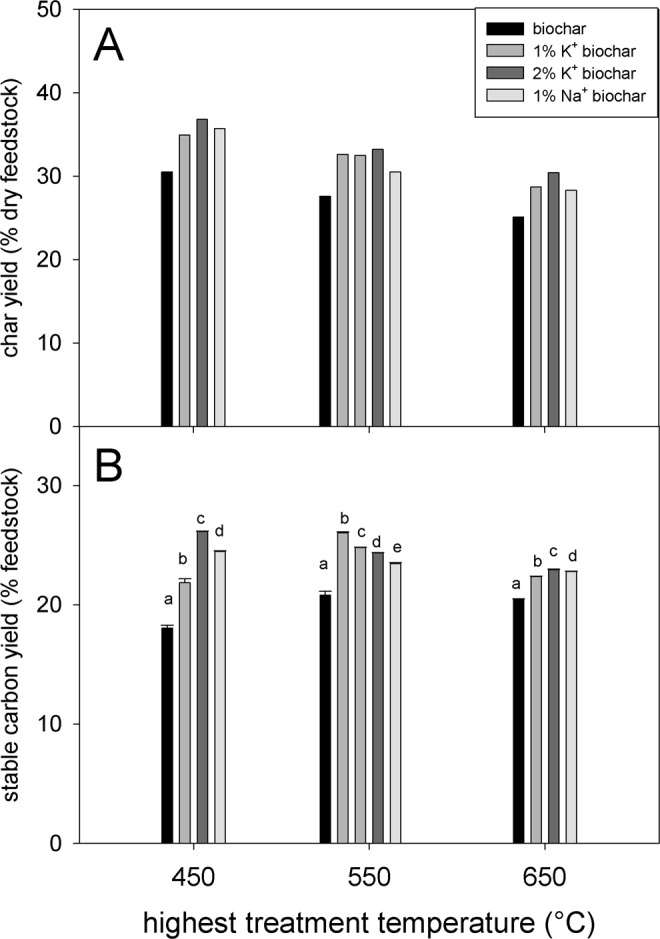
*Miscanthus* biochar yield (**A**; n = 1), and stable carbon yield (**B**; n = 3) of 13 biochar, produced at three different temperatures with four different AM dopings (1% K^+^ doped biochar produced in duplicates at 550 °C). Error bars in Figure B show one standard deviation. The letters indicate statistically significant differences between the treatments, using one-way ANOVAs followed by Tukey post-hoc test on biochar produced at the same temperatures. No statistical analysis was performed on biochar yield (no replications of pyrolysis itself).

**Figure 3:**
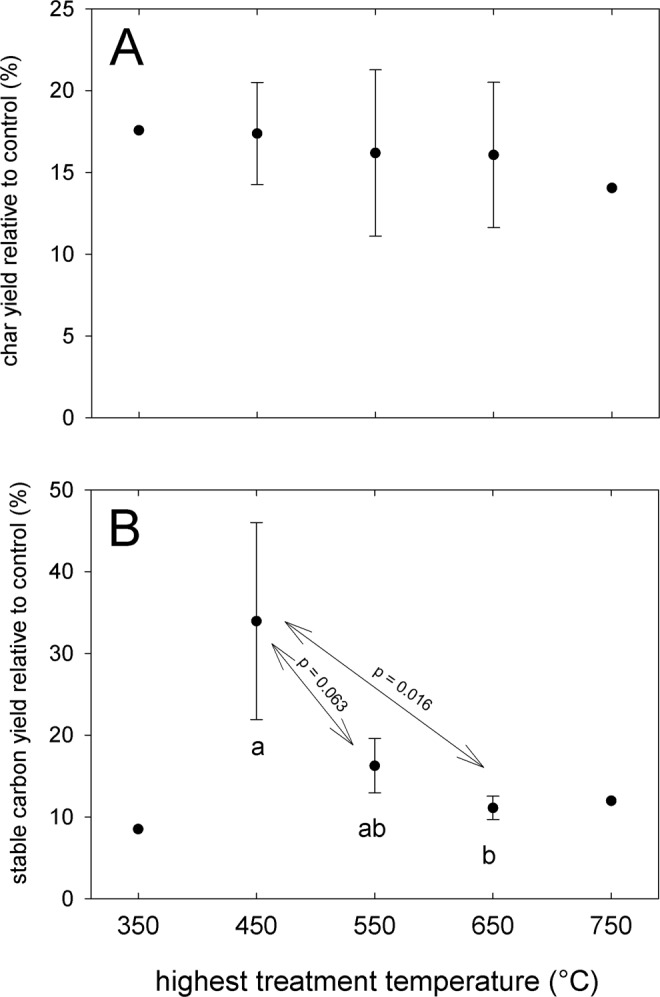
Biochar yield (**A**) and stable carbon content (**B**) of the biochar with AM doping relative to the control (biochar without doping). The error bars show one standard deviation. Letters show statistically significant differences between the treatments, with the respective p-values indicated above arrows. Number of n: 350 = 1 (only 1% K^+^), 450 = 3 (1% K^+^, 2% K^+^, and 1% Na^+^), 550 = 4 (1% K^+^ in duplicate, 2% K^+^, and 1% Na^+^), 650 = 3 (1% K^+^, 2% K^+^, and 1% Na^+^), 750 = 1 (1% K^+^).

Our results show that, as expected based on previous research and published literature, the biochar yield of *Miscanthus* biomass was strongly affected by the presence of AMs. Doping with AMs (1 wt% K^+^, 2 wt% K^+^ and 1 wt% Na^+^) unequivocally resulted in higher biochar yields, by 10.5–21.1% relative to the untreated biomass control, in the whole temperature range tested (350–750 °C) (Figs 1A, 2A). The relative biochar yield change compared to un-amended biomass pyrolysis (Fig. 3A) shows that in the tested temperature range the biochar yield increase was independent of the HTT.

Besides biochar yield, the content of stable carbon and subsequently the yield of stable carbon are the other two important parameters in terms of biochar’s ability to sequester carbon efficiently. In this study the stable carbon content was determined using a hydrogen peroxide oxidation method calibrated so that it corresponds to approx. 100 years of ageing in soil^8,9,27^. The yield of stable carbon was determined as a product of biochar yield and stable carbon content. Both Na^+^ and K^+^ doping showed similar performance in increasing both biochar (Fig. 2A) and stable carbon (Fig. 2B) yields. The increase in biochar yield is a result of catalysis of the charring process by AM catalysts in the pyrolysis of lignocellulosic materials. In the presence of these metals the reaction process favours charring and dehydrating reactions, versus fragmentation and depolymerization pathways, in the primary decomposition of the holocellulosic fraction^28^. In addition, AMs also enhance dehydration, demethoxylation, decarboxylation, and biochar formation in lignin pyrolysis^29,30^.

All AM treatments considerably increased the stable carbon yield (Fig. 2C) compared to the untreated controls. The relative change was between + 9.5% (1% K^+^ 650 °C) and +45.0% (2% K + 450 °C) (SI Table 4) and the average change was +20.4% for all AM treatments and pyrolysis temperatures.

This finding was confirmed with a different biomass feedstock material. Willow chips treated in the same way with 1% w/w K^+^ were also pyrolysed at 350, 550 and 750 °C (described in SI). The stable carbon yield was highest at a HTT of 550 °C with a 31.8% higher yield relative to the feedstock biomass than the corresponding untreated willow sample (SI Table 3). This shows that the effect of AM doping on stable carbon yield is not specific to one type of biomass (*Miscanthus)*.

### 450 °C pyrolysis maximises the carbon sequestration potential of miscanthus biochar

Tailoring the pyrolysis conditions and hence the biochar properties to suit the biochar end use is a profound and essential feature of biochar production, and therefore any effects of AM additives on available range of processing parameters are of high importance.

In general, both AMs performed comparably in terms of stable carbon yield when added at the same concentration (1wt %) (Fig. 2B). Increased AM loading reduced the HTT at which the highest stable carbon yield was obtained. While pyrolysis of *Miscanthus* loaded with 1% K^+^ yielded most stable carbon at 550 °C, in the case of *Miscanthus* loaded with 2% K^+^ this was observed at 450 °C (Fig. 2B). At higher temperatures these differences between AM loading levels became less pronounced (Fig 1B, 2B).

Taking all AM-treatments together the relative increase in stable carbon yield was highest at 450 °C (Fig. 3B) with +33.9% on average for both AM doping levels. This observation has very important practical implications, as it means that production units operating at the lower end of usual pyrolysis temperature ranges (450–500 °C) could be used. Such units would not require high-grade stainless steel as a material for construction, reducing costs compared to units specified for higher temperatures. With increasing pyrolysis temperature, the pH and surface area of biochar increase while at the same time the functionality (O/C, H/C ratios) decreases^31–33^. Therefore, producing biochar at moderate pyrolysis temperatures, i.e. 450–550 °C, is a good compromise, not only providing high yield of stable carbon, but also yielding biochar with beneficial properties for soil amelioration (high surface area, high CEC).

### AMs affect biochar microstructure and carbon stability

The increased stability of carbon can be explained by the catalytic effect of AMs on biomass pyrolysis, such as enhanced cross-linking reactions (e.g., dehydration forming C=C or C-O-C) resulting in a highly cross-linked biochar, compared to biochar from untreated biomass^34,35^.

To further corroborate these findings, the structure of the biochar was investigated using Raman spectroscopy and X-ray diffraction (XRD), focusing on differences in the biochar carbon structure resulting from K^+^ doping. Raman spectra of the six biochar types investigated are shown in SI Fig. 2. Meaningful features of the spectra are (i) the position of the G peak, (ii) the relative intensity of the D and G peaks (the so-called ID/IG ratio), (iii) the shift of the D peak, and (iv) the width of the peaks. SI Fig. 2 shows that for both series the increase of temperature leads to a) a shift of the D-peak to lower wavenumbers, indicating a slight reduction in the size of defect free and/or edge free regions, b) a decrease in the width of the D peaks with increasing temperature, indicating a reduction in the structural disorder. At any given process temperature, the use of K^+^ (1 and 2 wt%) leads to lower G peak intensities, i.e. to higher ID/IG ratio. This indicates that the amount of disordered material as determined by Raman spectroscopy is larger for K^+^ treated *Miscanthus* biochar.

The XRD analysis of *Miscanthus* and K^+^ *Miscanthus* biochar confirmed the differences in biochar structure resulting from doping with potassium (see SI Table 5). The content of graphitic carbon, although small, increases because of K^+^ doping at all temperatures, with the effect being strongest at the lower end of the temperature range investigated. This is comparable with the trend seen for yield of stable carbon.

Based on this evidence we can conclude that although the carbon structure in the K^+^ *Miscanthus* biochar appears to be less ordered and more defective than that of untreated *Miscanthus* biochar, it has a comparable recalcitrance. It is important to note here that in comparing the information obtained from Raman spectroscopy and XRD care has to be taken since XRD is a bulk technique while Raman is sampling a few hundred nanometres close to the surface. Hence findings from these two techniques must be looked at as complementary. The overall effect of AM on biochar and stable carbon yield is schematically shown in SI Fig. 6. This finding is fundamental for developing biochar for carbon sequestration purposes as it means that use of AM doping can make pyrolysis much more efficient in converting biomass carbon to stable biochar carbon.

### AMs doping enhances biochar’s role as a carbon sequestration tool

The potential of biochar to store atmospheric carbon has been estimated to be between 0.7–1.8 Gt CO_2_-C(eq) yr^−1^^3,12,13^. Assuming a stable carbon increase of 45% as observed in this study for 2% potassium addition increases the carbon sequestration potential of biochar to between 1 and 2.6 Gt CO_2_-C(eq) yr^−1^, which corresponds to over 7% of current annual GHG emissions^36^ and close to 25% of annual atmospheric CO_2_ removal necessary to maintain atmospheric concentrations of CO_2_ at safe levels^37^.

Another conclusion that can be drawn from this finding is that to sequester a given amount of carbon in form of biochar, 31% less biomass, and therefore land would be required, compared to biochar without AM additives. This would further reduce biochar’s already relatively low land requirement, compared to other NETs options^13^.

In addition, tests of the K^+^-enhanced biochar showed that the majority of the K^+^ contained in the biochar was available and could be utilised by plants upon application of the amended biochar to soil (based on 0.01 M CaCl_2_-extraction (SI Table 6)). Therefore, K^+^-enhanced biochar provides not only a better tool for carbon sequestration, but also a better slow-release K-fertiliser, compared to unamended biochar.

To date, viable biochar applications have been inextricably linked to biochar’s ability to increase crop yields^5,38^. Due to its much higher carbon sequestration potential, AM-enriched biochar offers a price competitive option for atmospheric carbon removal compared to bioenergy and carbon capture and storage (BECCS), and direct air capture (DAC) etc., especially if low-cost sources of potassium, such as ash^39^, can be utilised. Under such scenario, crop growth improvements would still be a desirable option^40^, in fact a more likely one due to the increased K content, but no longer as critical for viable biochar implementation. This opens-up a whole new range of potential applications where carbon sequestration can be achieved, but where only small or no crop yield benefits could be expected, e.g., fertile soils, contaminated land, as well as non-soil applications (e.g., building materials, underground storage). This is a breakthrough approach to biochar deployment and a paradigm shifting finding.

## Methods

### Biochar production

To prepare AM-loaded *Miscanthu*s with 1% and 2% w/w K^+^ content (0.256 and 0.513 mmol g^−1^ dry feedstock), potassium was added in a similar manner to previous work^23,41,42^ by spraying an aqueous solution (174.7 g L^−1^ and 349.5 g L^−1^) of potassium acetate (Sigma-Aldrich, ≥99.0%) onto oven-dried *Miscanthus* chips (approximately 10 mm in size) spread in a thin layer. In this way the desired level of potassium was introduced, and the biomass moisture was restored to the original level of ~12 wt%. Similarly, Na^+^ in form of an aqueous solution of sodium acetate (anhydrous, 98% pure, Fisher Scientific) (146 g L^−1^ sodium acetate) was applied to *Miscanthus* chips to introduce the same concentration in moles per weight of *Miscanthus* as 1% (w/w) K^+^ (256.4 μmol g^−1^ dry feedstock). This ensures that the same molar amount of AM is present in the 1% AM treatments independently of their molar weight which is equivalent to 0.59% (w/w) Na^+^. These organic salts were selected as additives due to their easy applicability, relatively low costs (potassium acetate 650–850 USD t^−1^^43^), bulk availability, widespread use (e.g., de-icing, drilling muds, fertilisers, etc.), and low environmental impact^44,45^.

The untreated *Miscanthus* samples were pyrolysed at 350, 450, 550, 650 and 750 °C with a continuous auger reactor described in Buss *et al*.^21^ with mean residence time of the biomass in the heated zone of around 21.5 minutes. The 1% K^+^ doped *Miscanthus* was pyrolysed in the same temperature range, but with duplicate at 550 °C. *Miscanthu*s doped with the other two AM treatments (2% K and 1% Na) was pyrolysed at the HTTs of 450 °C, 550 °C and 650 °C.

### Biochar carbon stability

In this work, the chemical stability of biochar against oxidation in the environment was tested using a hydrogen peroxide wet oxidation method^9^. This method has been calibrated on naturally aged charcoal samples and corresponds to 92 and 187 years at mean annual temperatures of 17 °C and 7 °C, respectively. Hence, the stable carbon content determined in this study reflects the amount of carbon that is sequestered in the soil and for which carbon credits could be given. The method has also been cross-referenced with other proxies for carbon stability^8^, such as fixed carbon determined by proximate analysis (comparison of the two methods on the biochar samples from this study is provided in the supplementary information). The method is described in Cross *et al*.^9^ and in brief here. Before analysis, all biochars were ground to fine powder using a pestle and mortar, and homogenised sub-samples were used for the test. A char sample containing 0.1 g of C was mixed in a test tube with 0.01 mol of H_2_O_2_ in 7 mL of DI water. The test tubes were subsequently heated to 80 °C and kept at this temperature, with occasional agitation, for two days until the solution had evaporated. Subsequently, the remaining material was dried overnight at 105 °C before weighing the amount of residual char, and C content analysis. The carbon amount prior and post ageing was then calculated using the char amount and carbon content. The ratio of retained C to initial C gives the % stable carbon content.

$$stable\,carbon\,content( \% )=\frac{residual\,char\,mass\,\ast \,residual\,char\,carbon\,content}{initial\,char\,mass\,\ast \,initial\,char\,carbon\,content}$$

The analysis was performed in triplicate. By multiplying the stable carbon content by the char yield the stable carbon yield was calculated as described for the fixed carbon content by Antal and Grønli (2003)^18^. This corresponds to the efficiency of carbon conversion from biomass to biochar and serves as the key parameter for comparison in this work.

### Raman spectroscopy

Raman spectra measurements were performed using a Renishaw InviaH instrument equipped with a green laser source (wavelength: 514.5 nm). All samples were taken as received (crushed) and placed on a microscope glass slide and flattened to obtain an optical field as flat as possible to improve the focus on the sample. Several points were examined for each sample. For each point we started with a low magnification objective (5x) zooming-in using a 20x and finally recording spectra with a 50x objective. The area of each spot examined had a width of about 2 μm^2^. Raman analysis being a volume technique, the signal is gathered from the sample surface up to a depth that varies with the optical properties of the analysed materials. In sp2-rich carbon materials this depth is of a few hundred nanometres (Ni, Z., Wang, Y., Yu, T. *et al*. Raman spectroscopy and imaging of graphene Nano Res. (2008) 1: 273. doi.org/10.1007/s12274–008–8036–1). The laser power was set at 5 mW in order to obtain a good signal-to-noise ratio while avoiding damage of the sample due to excessive heating. Each measurement was carried out in extended mode (100 cm^−1^ to 3500 cm^−1^) with an exposure time of 10 s and with 3 accumulations.

### X-ray diffraction (XRD)

A Bruker D8 advance XRD instrument with a Cu Anode was used at 40 mA and 40 kV with a NaI detector which analysed at 1.5 s/step from 2 to 65° in 0.025°/step increments. The raw data were evaluated with the software TOPAS 3.0 Rietveld analysis. The biochar samples were spiked with 20% calcite to quantitatively measure the composition of mineral in the samples relative to a known concentration of calcite.

### Statistics

Two-sample, two-sided, equal variances t-tests in Microsoft Excel were performed to determine effects of the 1% K^+^ addition on stable carbon yields. One-way ANOVAs followed by Tukey post-hoc tests (in SigmaPlot 13.0) were used to investigate the effect of the various AM treatments on stable carbon yield and the effect of the HTT on char yield and stable carbon yield taking the AM treatments as replications. Significant differences are given with a significance level of p < 0.05.

## Supplementary information

Supplementary information

## Data Availability

All data related to this experiment are provided within the article and the Supplementary Information.

## References

[1] IPCC 2014: Summary for Policymakers. Climate Change 2014: Mitigation of Cliamte Change. Contribution of Woeking group III to the Fifth Assessment Report of the Intergovernmental Panel on Climate Change [Edenhofer, O., R. Pichs-Madruga, Y. Sokona, E. Farahani, S. Kadner, K. Seyboth, A. Adler,. *mbridge Univ*. *Press*. *Cambridge*, *United Kingdom New York*, *NY*, *USA*. (2014).

[2] Fuss S (2014). Betting on negative emissions. Nat. Clim. Chang..

[3] Woolf D, Amonette JE, Street-Perrott FA, Lehmann J, Joseph S (2010). Sustainable biochar to mitigate global climate change. Nat. Commun..

[4] Lehmann J (2007). A handful of carbon. Nature.

[5] Woolf D, Lehmann J, Lee DR (2016). Optimal bioenergy power generation for climate change mitigation with or without carbon sequestration. Nat. Commun..

[6] Wang, J., Xiong, Z. & Kuzyakov, Y. Biochar stability in soil: Meta-analysis of decomposition and priming effects. *GCB Bioenergy* 1–12 10.1111/gcbb.12266 (2015).

[7] Lehmann, J. *et al*. Chapter 10: Persistence of biochar in soil. in *Biochar for Environmental Management: Science and Technology and Implementation*, *second Edition* 169–182 (Earthscan Ltd., London 2015).

[8] Crombie K, Mašek O, Sohi SP, Brownsort P, Cross A (2013). The effect of pyrolysis conditions on biochar stability as determined by three methods. GCB Bioenergy.

[9] Cross A, Sohi SP (2013). A method for screening the relative long-term stability of biochar. GCB Bioenergy.

[10] Budai, A. *et al*. *International Biochar Initiative: Biochar Carbon Stability Test Method: An assessment of methods to determine biochar carbon stability*. (2013).

[11] IPCC. *Chapter 8: Climate Change 2013: The physical science basis*. *Contribution of working group I to the fifth assessment report of the intergovernmental panel on climate change*10.1017/CBO9781107415324 (2013).

[12] Paustian K (2016). Climate-smart soils. Nature.

[13] Smith P (2016). Soil carbon sequestration and biochar as negative emission technologies. Glob. Chang. Biol..

[14] Zhang Q, Chang J, Wang T, Xu Y (2007). Review of biomass pyrolysis oil properties and upgrading research. Energy Convers. Manag..

[15] Crombie K, Mašek O (2014). Investigating the potential for a self-sustaining slow pyrolysis system under varying operating conditions. Bioresour. Technol..

[16] Crombie K, Mašek O (2015). Pyrolysis biochar systems, balance between bioenergy and carbon sequestration. GCB Bioenergy.

[17] Liu Z, Demisie W, Zhang M (2013). Simulated degradation of biochar and its potential environmental implications. Environ. Pollut..

[18] Antal MJ, Grønli M (2003). The art, science, and technology of charcoal production. Ind. Eng. Chem. Res..

[19] Ameloot N, Graber ER, Verheijen FGA, De Neve S (2013). Interactions between biochar stability and soil organisms: Review and research needs. Eur. J. Soil Sci..

[20] Evangelou MWH, Conesa HM, Robinson BH, Schulin R (2012). Biomass production on trace element–contaminated land: a review. Environ. Eng. Sci..

[21] Buss W, Graham MC, Shepherd JG, Mašek O (2016). Suitability of marginal biomass-derived biochars for soil amendment. Sci. Total Environ..

[22] Wang Z, Wang F, Cao J, Wang J (2010). Pyrolysis of pine wood in a slowly heating fixed-bed reactor: Potassium carbonate versus calcium hydroxide as a catalyst. Fuel Process. Technol..

[23] Fuentes ME (2008). A survey of the influence of biomass mineral matter in the thermochemical conversion of short rotation willow coppice. J. energy Inst..

[24] Richards GN, Shafizadeh F, Stevenson TT (1983). Influence of sodium chloride on volatile products formed by pyrolysis of cellulose: Identification of hydroxybenzenes and 1-hydroxy-2-propanone as major products. Carbohydr. Res..

[25] Zhao L (2017). Roles of Phosphoric Acid in Biochar Formation: Synchronously Improving Carbon Retention and Sorption Capacity. J. Environ. Qual..

[26] Ren N, Tang Y, Li M (2018). Mineral additive enhanced carbon retention and stabilization in sewage sludge-derived biochar. Process Saf. Environ. Prot..

[27] Masek O, Brownsort P, Cross A, Sohi S (2013). Influence of production conditions on the yield and environmental stability of biochar. Fuel.

[28] Di Blasi C (2009). Combustion and gasification rates of lignocellulosic chars. Prog. Energy Combust. Sci..

[29] Jakab E, Faix O, Till F (1997). Thermal decomposition of milled wood lignins studied by thermogravimetry/mass spectrometry. J. Anal. Appl. Pyrolysis.

[30] Kleen M, Gellerstedt G (1995). Influence of inorganic species on the formation of polysaccharide and lignin degradation products in the analytical pyrolysis of pulps. J. Anal. Appl. Pyrolysis.

[31] Harvey OR, Herbert BE, Rhue RD, Kuo LJ (2011). Metal interactions at the biochar-water interface: Energetics and structure-sorption relationships elucidated by flow adsorption microcalorimetry. Environ. Sci. Technol..

[32] Jindo K, Mizumoto H, Sawada Y, Sonoki T (2014). Physical and chemical characterization of biochars derived from different agricultural residues. Biogeosciences.

[33] Ronsse F, van Hecke S, Dickinson D, Prins W (2013). Production and characterization of slow pyrolysis biochar: influence of feedstock type and pyrolysis conditions. GCB Bioenergy.

[34] Le Brech Y (2016). Effect of Potassium on the Mechanisms of Biomass Pyrolysis Studied using Complementary Analytical Techniques. ChemSusChem.

[35] Liu D, Yu Y, Long Y, Wu H (2015). Effect of MgCl2 loading on the evolution of reaction intermediates during cellulose fast pyrolysis at 325 °C. Proc. Combust. Inst..

[36] The European Academies’ Science Advisory Council. *EASAC policy report 35: Negative emission technologies*: *What role in meeting Paris Agreement targets?* (2018).

[37] EASAC. *Science Advice for the Benefit of Europe Negative emission technologies: What role in meeting Paris Agreement targets? EASAC Policy Report* (2018).

[38] Peters, J., Iribarren, D. & Dufour, J. Biomass pyrolysis for biochar or energy applications? A life cycle assessment. *Environ*. *Sci*. *Technol*. 150401141039001, 10.1021/es5060786 (2015). 10.1021/es5060786 25830564

[39] Buss, W., Jansson, S., Wurzer, C. & Mašek, O. Synergies between BECCS and biochar - maximizing carbon sequestration potential by recycling wood ash - accepted. *ACS Sustain*. *Chem*. *Eng*. 10.1021/acssuschemeng.8b05871 (2019).

[40] Jeffery S, Verheijen FGa, van der Velde M (2011). & Bastos, a. C. A quantitative review of the effects of biochar application to soils on crop productivity using meta-analysis. Agric. Ecosyst. Environ..

[41] Nowakowski DJ, Jones JM (2008). Uncatalysed and potassium-catalysed pyrolysis of the cell-wall constituents of biomass and their model compounds. J. Anal. Appl. Pyrolysis.

[42] Nowakowski DJ, Jones JM, Brydson RMD, Ross AB (2007). Potassium catalysis in the pyrolysis behaviour of short rotation willow coppice. Fuel.

[43] Alibaba.com. www.alibaba.com/showroom/potassium-acetate-price.html*accessed 20/11/2017* (2017).

[44] Fischel, M. *Evaluation of selected deicers based on a review of the literature*, *Report No*. *CDOT-DTD-R-2001-15*, *Colorado Department of Transportation*, *Research Branch*. (2001).

[45] EPA. Environmental Impact and Benefit Assessment for the Final Effluent Limitation Guidelines and Standards for the Airport Deicing Category. 1–187 (2012).

