# Supplementary material for: Potassium doping increases biochar carbon sequestration potential by 45%, facilitating decoupling of carbon sequestration from soil improvement
Source: Sci Rep. 2019 Apr 2;9:5514. doi: 10.1038/s41598-019-41953-0 (PMC6445287; doi:10.1038/s41598-019-41953-0)
Supplement: Supplementary file 1 — Supplementary information [file 41598_2019_41953_MOESM1_ESM.pdf]

Supplementary Information

Potassium doping increases biochar carbon sequestration potential by 45%, facilitating decoupling of carbon sequestration from soil improvement

Ondřej Mašek[a*], Wolfram Buss[a], Peter Brownsort[b], Massimo Rovere[c], Alberto Tagliaferro[c], Ling Zhao[d], Xinde Cao[d], and Guangwen Xu[e]

1 Materials and methods

1.1 Doping of willow chips with potassium acetate

Willow chips were amended with 1% K^+ in the form of potassium acetate as discussed for *Miscanthus* in the material and methods section of the main manuscript and pyrolysed at 350, 550 and 750°C.

1.2 Washing of *Miscanthus* to produce low-ash feedstock

To obtain low-ash biomass, the chips were washed twice in hot tap water and once with cold DI water over night similar to Di Blasi et al. (2000) and Eom et al. (2011).

1.3 Ultimate analysis

The relative contents of C, H and N were analysed in duplicates using an elemental analyser (Flash 2000; CE Elantech Inc, Lakewood, NJ, USA) at London Metropolitan University (London, UK). The O content was calculated by difference (100-C-H-N-ash).

1.4 Proximate analysis

Proximate analysis is a method frequently used for basic characterisation of char sample which differentiates between moisture, volatile matter, fixed carbon and ash content [3]. This is a simple analysis to determine differences in stability of the organic matter against thermal degradation which has been used as a proxy for carbon stability [4].

Proximate analyses were performed, using a Mettler-Toledo TGA/DSC1 [5] on approx. 15 mg samples in 70 µl alumina crucibles. The temperature profile used was as follows: heating under N_2 (oxygen free) up to 110°C at a heating rate of 25°C min^{-1}, holding at 110°C for 10 min (moisture removal), heating up to 900°C at a heating rate of 25 °C min^{-1} and holding at

900°C for 10 min (volatile matter content). Finally, while still at 900°C the purge gas is switched to air and the sample is combusted (for 20 min) to determine the fixed carbon and ash content of the sample. The analyses were performed in triplicates.

The fixed carbon content was then calculated on dry, ash-free basis. Additionally, by multiplying the fixed carbon content by the char yield the fixed carbon yield was calculated as described in Antal et al. (2003) [3] which corresponds to the efficiency of carbon conversion. This method was used to correlate with the stable carbon method described and used in the main manuscript.

2 Results and Discussions

Following Tables and Figures complement the results already present in the main manuscript (SI Table 1, 4) and present new data for biomass washing prior to pyrolysis (SI Table 2), another feedstock material (SI Table 3), more detailed analysis of the structure of the biochar (SI Figure 2, 3, 4, 5 and SI Table 5) and $CaCl_2$-extractable nutrients (SI Table 6). SI Figure 1 supports the validity of the hydrogen peroxide oxidation as a method for carbon stability by comparing it with data from fixed carbon from proximate analysis.

SI Table 1: Characteristics of biochar produced from *Miscanthus* chips. Displayed are the pyrolysis highest treatment temperature (HTT), results of CHN(O) (ultimate) analysis (mean of duplicates) and proximate analysis (mean and standard deviation of triplicates on dry, ash-free basis (daf)). 1% K^+ 550 was produced in duplicates. VM = Volatile Matter as determined by proximate analysis.

HTT	treatment	char yield % dry	C %	H %	N %	O* %	O:C w/w	H:C w/w	O:C mol/mol	H:C mol/mol	ash %dry AV	±	SD	VM % daf AV	±	SD	fixed C % daf AV	±	SD
350	untreated	40.4	73.85	3.62	0.1	22.43	0.30	0.05	0.46	0.59	6.0	±	2.3	35.1	±	0.9	64.9	±	0.9
	1% K^+ addition	47.5	67.32	3.45	0.10	29.14	0.43	0.05	0.65	0.61	9.3	±	1.8	34.3	±	0.7	65.7	±	0.7
450	untreated	30.5	69.88	2.28	0.01	27.83	0.40	0.03	0.60	0.39	11.4	±	1.9	25.0	±	0.4	75.0	±	0.4
	1% K^+ addition	34.9	73.88	2.08	0.01	24.04	0.33	0.03	0.49	0.34	11.2	±	0.7	23.2	±	0.5	76.8	±	0.5
	2% K^+ addition	36.8	72.32	2.60	0.01	25.08	0.35	0.04	0.52	0.43	10.9	±	1.7	26.1	±	1.7	73.9	±	1.7
	Na^+ addition	35.7	72.66	2.34	0.01	25.00	0.34	0.03	0.52	0.39	10.2	±	1.0	24.5	±	1.1	75.5	±	1.1
550	untreated	27.6	82.27	2.13	0.01	15.60	0.19	0.03	0.28	0.31	10.9	±	1.2	14.3	±	2.0	85.5	±	1.8
	1% K^+ addition	32.6	76.05	1.57	0.01	22.37	0.29	0.02	0.44	0.25	11.1	±	4.1	14.7	±	1.5	85.3	±	1.5
	1% K^+ addition	32.5	75.1	1.5	0.1	12.2	0.16	0.02	0.24	0.25	10.8	±	0.5	17.2	±	1.7	82.8	±	1.7
	2% K^+ addition	33.2	74.45	1.39	0.01	24.16	0.32	0.02	0.49	0.22	13.3	±	0.5	20.1	±	0.7	79.9	±	0.7
	Na+ addition	30.5	77.57	2.11	0.01	20.32	0.26	0.03	0.39	0.33	10.2	±	1.2	15.8	±	1.3	84.2	±	1.3
650	untreated	25.1	82.00	1.03	0.01	16.97	0.21	0.01	0.31	0.15	9.7	±	0.8	8.3	±	0.1	91.7	±	0.1
	1% K^+ addition	28.7	78.04	0.83	0.01	21.13	0.27	0.01	0.41	0.13	12.7	±	0.8	13.6	±	0.9	86.4	±	0.9
	2% K^+ addition	30.4	75.66	1.27	0.01	23.07	0.30	0.02	0.46	0.20	14.4	±	1.0	18.2	±	0.6	81.8	±	0.6
	Na^+ addition	28.3	80.05	1.24	0.01	18.71	0.23	0.02	0.35	0.19	11.3	±	2.7	11.5	±	1.4	88.5	±	1.4
750	untreated	23.5	83.48	0.87	0.10	15.56	0.19	0.01	0.28	0.13	11.4	±	0.9	7.5	±	0.7	92.5	±	0.7
	1% K^+ addition	26.8	74.37	0.67	0.10	24.87	0.33	0.01	0.50	0.11	14.6	±	0.2	12.6	±	0.2	87.4	±	0.2

* determined by difference

SI Table 2: Char and stable carbon yield of *Miscanthus* pyrolysed at three different temperatures. The biomass was either treated by washing with tap water or 1% (w/w) K^+ in the form of K acetate was added.

	350°C	550°C	750°C
	char yield (dry basis)		
washed	42.9	26.1	21.4
untreated	40.4	28.8	23.5
1% K^+ addition	47.5	32.6	26.8
	stable carbon yield (% daf feedstock)		
washed	20.6	18.4	19.4
untreated	21.9	21.9	20.9
1% K^+ addition	24.7	29.3	24.3

SI Table 3: Char and stable carbon yield of Willow pyrolysed at three different temperatures. The biomass was either untreated or 1% (w/w) K^+ in the form of K acetate was added.

	350°C	550°C	750°C
	char yield (dry basis)		
untreated	43.9	26.2	21.3
1% K^+ addition	43.1	29.6	22.1
	stable carbon yield (% daf feedstock)		
untreated	17.8	17.9	18.6
1% K^+ addition	17.3	23.6	17.1

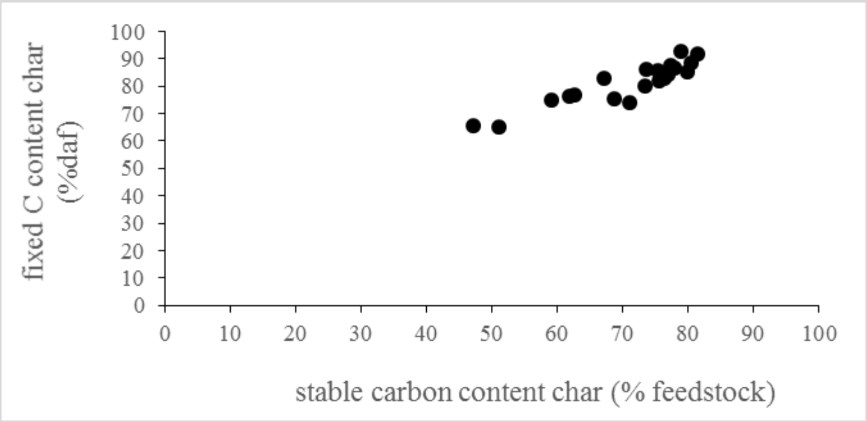

SI Figure 1: Correlation of fixed carbon content with stable carbon content of the 17 biochar. The Pearson Momentum Correlation coefficient is 0.924.

SI Table 4: Stable carbon content on dry basis in char and stable carbon yield (based on the feedstock, taking into account the char yield). Total value with average and standard deviation (n = 3) and relative changes compared to the control (untreated char) at the respective HTT (highest treatment temperature) displayed.

		**stable C content C (%dry)**		**stable C yield (%dry)**	
**HTT**	**treatment**	**total**	**relative change**	**total**	**relative change**
350	untreated	51.0 ± 0.6		20.6 ± 0.2	
	1% K+ addition	47.1 ± 0.9	-7.7%	22.4 ± 0.0	+8.5%
450	untreated	59.2 ± 0.8		18.0 ± 0.2	
	1% K+ addition	62.6 ± 1.0	+5.8%	21.9 ± 0.4	+21.1%
	2% K+ addition	71.1 ± 0.1	+20.2%	26.2 ± 0.0	+45.0%
	Na+ addition	68.6 ± 0.2	+16.0%	24.5 ± 0.1	+35.7%
550	untreated	75.4 ± 1.1		20.8 ± 0.3	
	1% K+ addition	76.3 ± 0.1	+1.2%	24.8 ± 0.0	+19.2%
	1% K+ addition	79.9 ± 0.0	+5.9%	26.0 ± 0.1	+25.1%
	2% K+ addition	73.4 ± 0.1	-2.7%	24.4 ± 0.0	+17.0%
	Na+ addition	76.9 ± 0.3	+1.9%	23.4 ± 0.1	+12.6%
650	untreated	81.4 ± 0.3		20.4 ± 0.1	
	1% K+ addition	78.0 ± 0.1	-4.2%	22.4 ± 0.0	+9.5%
	2% K+ addition	75.5 ± 0.2	-7.3%	23.0 ± 0.0	+12.3%
	Na+ addition	80.5 ± 0.2	-1.1%	22.8 ± 0.1	+11.5%
750	untreated	78.9 ± 0.3		18.5 ± 0.1	
	1% K+ addition	77.4 ± 0.4	-1.8%	20.7 ± 0.1	+12.0%

SI Table 5: Biochar structure as determined using XRD (values provided in wt%)

	Miscanthus 350	K^+ *Miscanthus* 350	*Miscanthus* 550	K^+ *Miscanthus* 550	*Miscanthus* 750	K^+ *Miscanthus* 750
Quartz	0.1	0.1	0.1	0.1	0.1	0.0
Graphitic C	0.0	0.3	0.1	0.2	0.5	0.6
Dolomite	2.4	1.2	1.4	2.2	1.4	2.1
Amorphous C	97.5	98.4	98.4	97.5	98.1	97.3

Raman analysis

Raman spectra of the six biochar batches investigated are shown in SI Figure 2. In these spectra, the Raman regions of interest (1000-1800 cm^{-1} and 2200-3400 cm^{-1}) are superimposed on a background due to photoluminescence. The intensity of the background decreases as T increases due to the loss of H and subsequent increase of non-radiative recombination paths [6]. However, the most interesting information come from the 1000-1800 cm^{-1} region where the so-called D and G peaks (a typical signature for sp2 hybridized carbon

[7] give their contribution. In SI Figure 2 A we report the superposition of the 1000-1800 cm^{-1} region of the Raman spectra for the *Miscanthus* biochar samples, while in SI Fig. 2 B the analogous information is reported for the K^+ treated samples. Spectra have been normalised to the D peak intensity in order to make below discussed features more evident. The lower wavenumber region is associated with the contribution of the D peak, generated by the presence of edge-like structures and defects [8]. The contribution to the higher wavenumber region is given by the G peak, associated to the stretching of sp2 carbon bonds [9]. Meaningful features of the spectra are (i) the position of the G peak, (ii) the relative intensity of the D and G peaks (the so-called ID/IG ratio), (iii) the shift of the D peak, and (iv) the width of the peaks. The position of the G peak and the widths of the peaks are determined by the type of local structure and the amount of structural disorder [10]. The D-peak position is determined by the volume of the 'grains' [11] and the ID/IG ratio in turn is affected by the density of edges and defects per unit volume [7]. Fig. 2 A and B evidence that for both series the increase of temperature leads to a) a shift of the D-peak to lower wavenumbers indicating a slight reduction in the size of defect free and/or edge free regions, b) a decrease in the width of the D peaks with increasing T, indicating a reduction in the structural disorder. Moreover, taking into account the value of the Full Width at Half Maximum of the G-peaks the analysis of Fig. 41 of J. Robertson 2002 indicates that the size of the 'grains' is around 1 nm[12].

In SI Figures 3, 4 and 5 the comparison of the Raman spectra of the *Miscanthus* and K^+ *Miscanthus* biochar are reported for each process temperature. At any given process temperature, the use of K^+ leads to lower G peak intensities, i.e. to higher ID/IG ratio. This indicates that the amount of disordered material is larger for K^+ treated *Miscanthus* biochar.

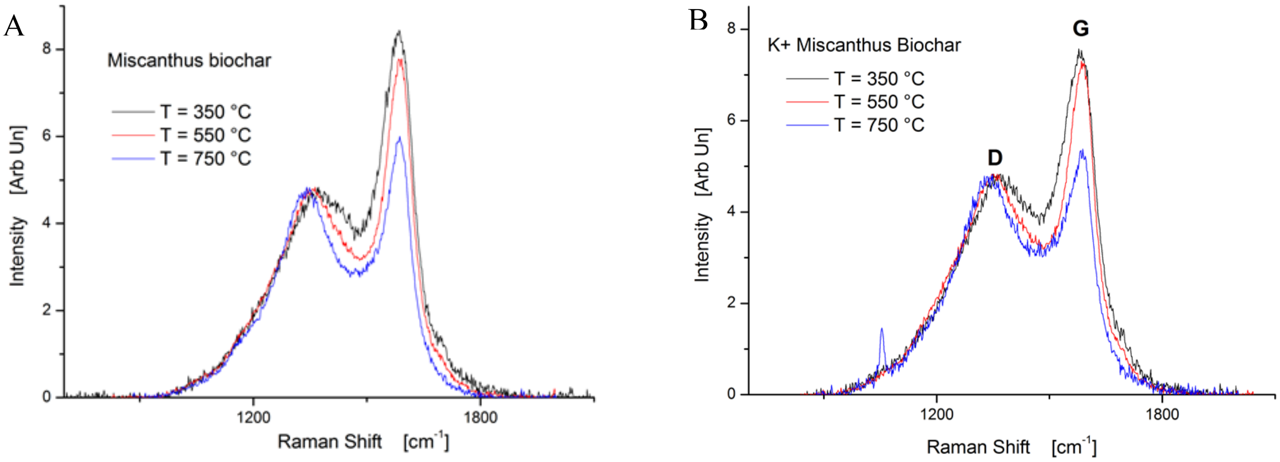

SI Figure 2: Raman signal in the D+G region for the two biochar sets (normalized at the D-peak height) produced at 350, 550 and 750 °C from raw (A) and 1% K^+ doped biomass (B)

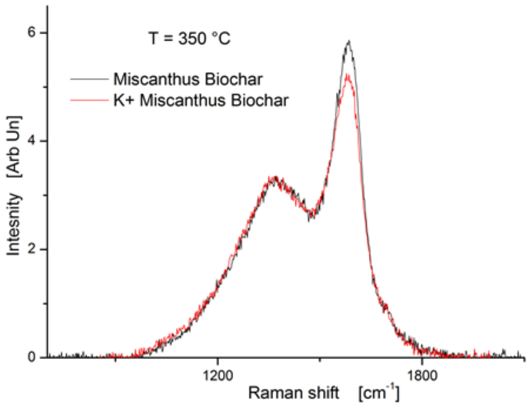

SI Figure 3 Comparison of T = 350 °C samples for the series ‘untreated’ and ‘1% K^+ doped’ biochar

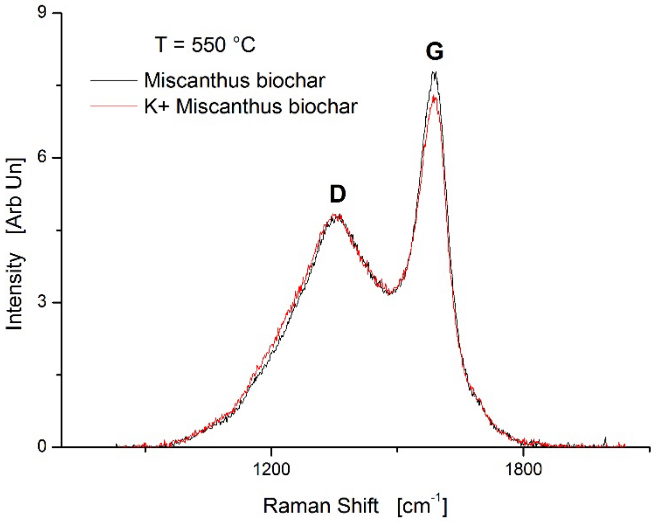

SI Figure 4 Comparison of T = 550 °C samples for ‘untreated’ and ‘1% K^+ doped’ biochar

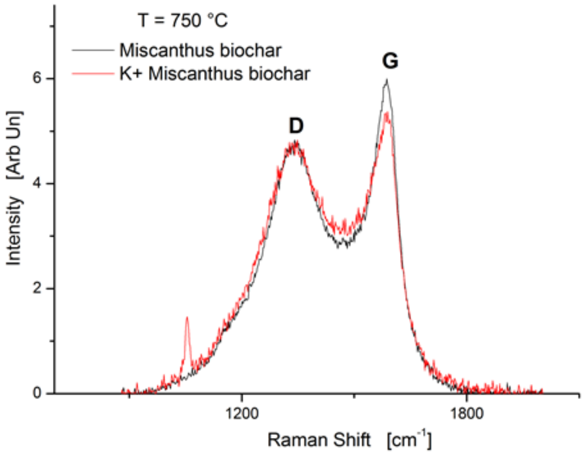

SI Figure 5 Comparison of T = 750 °C samples for the series ‘untreated’ and ‘1% K^+ doped’ biochar

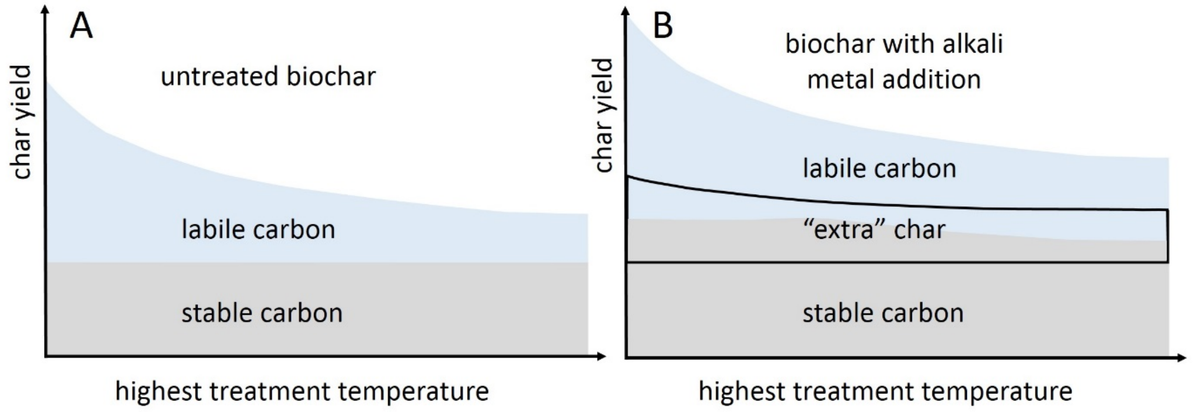

SI Figure 6: Schematic of the effect of addition of alkali metal prior to pyrolysis and pyrolysis temperature on char and stable carbon yield (corrected for additional ash introduced in form of alkali metal).

SI Table 6: Total and available concentration (%wt char) of K in untreated, 1% K-amended and 2% K-amended biochar produced at 550°C. Totals determined via modified dry ashing and available through extraction with 0.01 M $CaCl_2$.

	total K (%)		available K (%)		% available K of total K
	AV	SD	AV	SD	
untreated	0.72	0.02	0.20	0.01	27.1
1% K	2.53	0.13	1.29	0.04	50.7
2% K	4.35	0.12	2.77	0.03	63.6

References

1. Eom, I. Y. *et al.* Characterization of primary thermal degradation features of lignocellulosic biomass after removal of inorganic metals by diverse solvents. *Bioresour. Technol.* **102,** 3437–3444 (2011).
2. Di Blasi, C., Branca, C. & D'Errico, G. Degradation characteristics of straw and washed straw. *Thermochim. Acta* **364,** 133–142 (2000).
3. Antal, M. J. & Grønli, M. The art, science, and technology of charcoal production. *Ind. Eng. Chem. Res.* **42,** 1619–1640 (2003).
4. Crombie, K., Mašek, O., Sohi, S. P., Brownsort, P. & Cross, A. The effect of pyrolysis conditions on biochar stability as determined by three methods. *GCB Bioenergy* **5,** 122–131 (2013).
5. Buss, W. & Mašek, O. Mobile organic compounds in biochar - a potential source of contamination - phytotoxic effects on cress seed (Lepidium sativum) germination. *J. Environ. Manage.* **137,** 111–9 (2014).
6. Szatkowski, J. *et al.* Structural and photoluminescence study of diamond-like layers grown by electrochemical method. *Optoelectron. Adv. Mater. Rapid Commun.* **2,** 46–49 (2008).
7. Dresselhaus, M. S., Jorio, A., Souza Filho, A. G. & Saito, R. Defect characterization in graphene and carbon nanotubes using Raman spectroscopy. *Philos. Trans. R. Soc. A Math. Phys. Eng. Sci.* **368,** 5355–5377 (2010).
8. Pimenta, M. A. *et al.* Studying disorder in graphite-based systems by Raman spectroscopy. *Phys. Chem. Chem. Phys.* **9,** 1276–1290 (2007).
9. Sakata, H., Dresselhaus, G., Dresselhaus, M. S. & Endo, M. Effect of uniaxial stress on the Raman spectra of graphite fibers. *J. Appl. Phys.* **63,** 2769–2772 (1988).
10. Ferrari, A. C. & Robertson, J. Raman spectroscopy of amorphous, nanostructured, diamond-like carbon, and nanodiamond. *Philos. Trans. R. Soc. A Math. Phys. Eng. Sci.* **362,** 2477–2512 (2004).
11. Ferrari, A. C. & Basko, D. M. Raman spectroscopy as a versatile tool for studying the properties of graphene. *Nat. Publ. Gr.* **8,** 235–246 (2013).
12. Robertson, J. Diamond-like amorphous carbon. *Mater. Sci. Eng. R Reports* **37,** 129–281 (2002).
